# Validation of the Japanese version of the Interoception Sensory Questionnaire for individuals with autism spectrum disorder

**DOI:** 10.1038/s41598-022-25883-y

**Published:** 2022-12-15

**Authors:** Chihiro Itoi, Yuta Ujiie, Kanae Matsushima, Kohske Takahashi, Masakazu Ide

**Affiliations:** 1 NTT Communication Science Laboratories, 3-1 Morinosato Wakamiya, Atsugi, Kanagawa 243-0198 Japan; 2 National Rehabilitation Center for Persons with Disabilities, 4-1 Namiki, Tokorozawa, Saitama 359-8555 Japan; 3 College of Contemporary Psychology, Rikkyo University, 1-2-26 Kitano, Niiza-shi, Saitama, 352-8558 Japan; 4 Research Organization of Open Innovation and Collaboration, Ritsumeikan University, 2-150 Iwakura-Cho, Ibaraki, Osaka 567-8570 Japan; 5 Faculty of Rehabilitation, Kansai Medical University, 18-89 Uyamahigashi-Cho, Hirakata, Osaka 573-1136 Japan; 6 College of Comprehensive Psychology, Ritsumeikan University, 2-150 Iwakura-Cho, Ibaraki, Osaka 567-8570 Japan

**Keywords:** Neuroscience, Cognitive neuroscience, Perception

## Abstract

The Interoception Sensory Questionnaire (ISQ) is a self-report instrument used to assess the characteristics of interoceptive processing in individuals with autism spectrum disorder (ASD). Previous studies have shown that scores of the ISQ are more appropriate than other subjective measures for evaluating difficulties in interoceptive processing in individuals with ASD. Yet, no prior research has demonstrated the validation of the ISQ in Japanese samples. This study attempted to validate the Japanese version of the ISQ (ISQ-J) by examining its psychometric properties. We confirmed the score distribution, internal consistency, and factor structure in Japanese samples. We also examined the relationships with other interoceptive questionnaires. In addition, we compared the scores of the ISQ-J between adolescents and adults with ASD participants and control participants. Results of confirmatory factor analyses showed that the reliability of the ISQ-J in adults with ASD reached an acceptable level of a one-factor structure with excellent internal consistency (α = 0.963). The result of the ISQ-J showed a significant positive correlation with the measure of awareness of interoceptive sensitivity for localized bodily states; on the other hand, a significant negative correlation was found with those integrated bodily states. In addition, the ISQ-J scores were significantly higher in the ASD group than in the control group. The current findings depend on self-report data (including a diagnosis of ASD) to measure validity constructs. Additionally, since the ISQ-J was surveyed in adults with ASD, it is unclear whether similar the results would be obtained if the ISQ-J were conducted with children. These results indicate the validity and reliability of the ISQ-J and provide a tool for assessing confusion of interoceptive information in Japanese adults with ASD.

## Introduction

Atypical sensory processing is experienced by nearly 90% of individuals with autism spectrum disorder (ASD)^1,2^. Most previous studies on atypical sensory processing in individuals with ASD have examined the characteristics of exteroceptive sensory processing, which refers to the processing of sensory stimuli from outside the body, such as vision and audition^3,4^. However, the sensory processing characteristic of ASD is found not only in external sensory information but also in internal body information. The perception of internal body information, which is called interoception, is the sensation of information within the body, such as the state of one's internal organs and body fluids, and contributes to allostasis by providing information on visceral processes (sensations of hunger, pain, temperature, thirst, etc.). Indeed, the latest diagnostic criteria for ASD include not only the differences in response to sensory stimuli, such as hypersensitivity, hypo-sensitivity, and sensory seeking, but also a seeming indifference to pain and body temperature^5^.

Previous studies of autobiographies^6^ and interviews^7^ on ASD have suggested that individuals with ASD are less responsive to internal body information. For example, one found that individuals with ASD are characterized by reduced awareness of thirst^6^. In addition, clinical implications include a high rate of comorbidity of physical disabilities such as gastrointestinal symptoms^7^ and migraine headaches^8^, in individuals with ASD^9^. From these results, it is crucial to intervene appropriately in the atypical processing of interoceptive information as well as in problems of mental health to maintain the psychological and physical health of individuals with ASD.

As tools to measure interoceptive sensitivity, some questionnaires have been developed, including the Body Perception Questionnaire (BPQ)^10^, Multidimensional Interoceptive Assessment (MAIA)^11^, and Body Awareness Questionnaire (BAQ)^12^. However, these questionnaires consist of questions mainly focusing on “awareness” of bodily signals, which cannot cover all the difficulties of interoceptive sensory processing in ASD. For example, people with ASD have difficulty perceiving internal bodily signals such as thirst and fatigue and knowing how to deal with them; on the other hand, they notice localized bodily symptoms (e.g., dry mouth, facial twitches, heartbeat, and sweating palms)^13^. Previous studies about subjective interoceptive sensitivity in individuals with ASD, with some studies showing high^14^ and others low^6,15^ results, and those results seem to be contradicted. Although previous studies have used the BPQ as a measure of interoceptive sensitivity, the BPQ alone might not have fully captured the characteristics of interoceptive sensory processing in people with ASD. Therefore, it would be appropriate to use an assessment tool focused on the confusion of interoceptive information processing specific to people with ASD. To address these issues, the Interoception Sensory Questionnaire (ISQ) was developed to assess the characteristics of interoceptive processing in individuals with ASD^16^. The ISQ is a 20-item self-reported questionnaire. This questionnaire is constructed by conducting semi-structured interviews with adolescents and adults with ASD, to capture ASD-specific difficulties and confusions in internal sensory processing and to assess differences in internal sensory processing with and without ASD^16^. The characteristics of ASD’s interoceptive sensory processing obtained from the semi-structured interview—“difficulty in identifying and describing interoceptive body signals unless extremely hypo- and hyper-responsive, reliance on external cues, and reduced emotional and motivational components to work on physical states”—are reflected in the ISQ items. Therefore, the ISQ is more appropriate than other subjective measures of interoceptive sensitivity to assess difficulties in processing interoceptive information in individuals with ASD; however, it has not yet been validated in Japan.

This study aimed to validate the Japanese version of the Interoception Sensory Questionnaire (ISQ-J) in Japanese samples that included individuals with and without ASD. In this study, we confirmed the score distribution, internal consistency, factor structure of the ISQ-J, and the difference in ISQ-J scores between individuals with and without ASD. In addition, we examined the relationships with other relevant individual traits surveyed in the Toronto Alexithymia Scale (TAS-20)^17,18^, Body Perception Questionnaire-Body Awareness (BPQ-BA)^10^, and multi-dimensional assessment of interoceptive awareness (MAIA)^11^. We hypothesized that (1) the one-factor structure would be acceptable in Japanese samples, consistent with previous studies^16,19^, (2) ISQ-J scores would correlate positively with the scores of TAS-20^17,18^ and negatively with the MAIA^11^ and BPQ^10^, and (3) ISQ-J scores would be significantly greater in ASD samples than in control samples, consistent with the original study^16^.

## Methods

### Participants

We recruited 63 ASD samples and 326 control samples online. Individuals with ASD were recruited via community members (14.3%) and via links advertised on various autism-specific websites with no incentive offered. Approximately 8.0% of the control participants were students, and no incentive as offered to community members. Additional recruitment was via an Internet research service, where participants were paid for their time. We matched the age and gender of ASD and control samples. Table 1 lists the characteristics of survey participants. Demographic data, including age, gender, years of education, autism diagnosis and age at autism diagnosis, and coexistence with other clinical disorders, were collected.

**Table 1:** Demographic information (N = 289).

Variable	ASD group (N = 63)	Control group (N = 226)
Males (N, %)	20 (31.7)	89 (37.1)
Females (N, %)	38 (60.3)	151 (62.9)
Gender unspecified (N, %)	5 (7.9)	N/A
Age (years, SD)	31.4 (6.9)	31.2 (4.2)
Age at autism diagnosis (years, SD)	23.5 (9.39)	N/A
Years of education (years, SD)	15.3 (2.53)	14.9 (5.9)
**Survey source**		
Students (N, %)	N/A	9 (2.8)
Community members (N, %)	9 (14.3)	17 (5.2)
Social network service community (N, %)	54 (85.7)	N/A
Internet research service (N, %)	N/A	200 (92.0)

All ASD samples had been previously diagnosed with autism by a suitably qualified professional (pediatrician or psychiatrist). Of the 63 participants in the ASD group, 17 had only a diagnosis of ASD, 29 had comorbidities of other developmental disorders, such as attention deficits hyperactivity disorder (ADHD), and 28 had comorbidities of psychiatric disorders, such as depression and anxiety.

The control samples included university students (aged more than 18 years) recruited from universities and adults recruited via an Internet research service (iBRIDGE Co., Tokyo, Japan). We initially recruited 326 participants, from which we selected 226 participants. Those who checked the box for ASD, had a mental or physical disease, or did not faithfully answer the questions (errors is checking the name of a designated color; same answers to all questions) were excluded. In total, we used data of 63 ASD samples and 226 control samples for the analysis.

### Measures

#### The ISQ-J

- Scale Development: The ISQ^16^ assesses interoceptive processing difficulty in a single domain (e.g., “I have difficulty understanding when I am hungry or thirsty”). The ISQ contains 20 items on a 7-point Likert scale ranging from “strongly disagree” to “strongly agree.” The total possible scores range from 20 to 140. The Cronbach’s alpha coefficient of the original ISQ is 0.96.
- Translation Process: The translation was done according to internationally accepted guidelines^21^ with permission from the original ISQ developers. First, two English-Japanese bilingual speakers translated items of the original version of the ISQ from English to Japanese. Three investigators checked the preliminary Japanese version and chose the Japanese translation of the ISQ. Two individuals with ASD answered the ISQ-J to assess the questionnaire’s comprehension from which we concluded that there was no problem understanding the translated terminology used in the questionnaire. Then, another English-Japanese bilingual speaker who was not given any information about the original English version of ISQ translated the Japanese into English and checked whether the Japanese and back-translated items corresponded in meanings to the original items of ISQ. The back-translated items were sent to the original ISQ developers to confirm its equivalence to the original English version of the ISQ. The final approved version of the translation was used in the present study.

#### MAIA

The Japanese version of the MAIA^20^ contains eight sub-scales. In this study, four of the eight sub-scales of the MAIA were used, consistent with the previous study^16^: attention regulation (e.g., “I can refocus my attention from thinking to sensing my body.”), emotional awareness (e.g., “I notice how my body changes when I feel happy/joyful.”), self-regulation (e.g., “I can use my breath to reduce tension.”) and body listening (e.g., “I listen to my body to inform myself about what to do”). The four sub-scales consist of 19 items in total. Items are asked on a 6-point Likert scale, ranging from 0 (Never) to 5 (Always). The total possible scores range from 0 to 95.

#### BPQ-BA

Although only the MAIA was used as the questionnaire on interoceptive sensitivity to examine validity in a previous study^16^, the BPQ-BA^10^ was also administered in the present study, which includes a questionnaire on awareness of interoceptive sensitivity specifically related to negative emotions.

The Japanese version of the BPQ-BA^21^ assesses interoceptive awareness in a one-factor structure (e.g., “My mouth is drying,” “How fast I am breathing”), which consists of 26 items on a 5-point Likert scale ranging from “Never” to “Always.” We used the total score in the correlation analysis with the ISQ. The total possible scores range from 26 to 130.

#### TAS-20

The Japanese version of the TAS-20^22^ contains three domains: difficulty in identifying feelings (e.g., “I am often confused about what emotion I am feeling”), difficulty in describing feelings (e.g., “I am able to describe my feelings easily”), and externally oriented thinking (e.g., “I find examination of my feelings useful in solving personal problems”) It consists of 20 items on a 5-point scale ranging from “Definitely disagree” to “Definitely agree.” The total possible scores range from 20 to 100.

#### Autism Quotient (AQ)

The Japanese version of the AQ^23^ assesses autistic traits in five domains: social skills (e.g., “I find it hard to make new friends”), communication (e.g., “I frequently find that I don’t know how to keep a conversation going”), attention to detail (e.g., “I often notice small sounds when others do not”), attention switching (e.g., “I prefer to do things the same way over and over again”), and imagination (e.g., “I find making up stories easy”). The AQ contains 50 items on a 4-point Likert scale: “definitely disagree,” “slightly disagree,” “slightly agree,” and “definitely agree.” We scored each item according to the original scoring method^24^ from 0 (“definitely disagree” or “slightly disagree”) to 1 (“definitely agree” or “slightly agree”); we also used reverse scoring, as necessary. The total possible scores range from 0 to 50.

Table 2 shows the results of the group comparisons for questionnaires (MAIA, BPQ-BA, TAS-20 and AQ).

**Table 2:** Means and standard deviations of scale scores for ASD and control group.

	ASD group	Control group	t-test (*p*-value)	Effect size (Cohens *d*)
BPQ (M/SD)	71.35 (17.11)	56.79 (22.24)	p < 0.001	0.69
**MAIA (M/SD)**				
Attention regulation	19.33 (8.21)	24.98 (8.96)	p < 0.001	0.64
Emotional awareness	16.78 (6.58)	17.49 (6.38)	p = 0.43	0.11
Self-regulation	10.70 (5.22)	14.05 (5.03)	p < 0.001	0.65
Body listening	8.13 (4.27)	10.12 (3.96)	p < 0.001	0.47
TAS (M/SD)	63.59 (11.26)	54.09 (9.83)	p < 0.001	0.86
**AQ (M/SD)**				
Total	35.38 (7.07)	23.05 (6.21)	p < 0.001	1.92
Social skill	7.95 (2.46)	5.01 (2.50)	p < 0.001	1.18
Attention switching	7.63 (1.58)	4.97 (1.81)	p < 0.001	1.51
Attention detail	6.29 (2.27)	4.72 (1.82)	p < 0.001	0.82
Communication	7.21 (2.00)	3.99 (2.07)	p < 0.001	1.57
Imagination	6.30 (2.15)	4.36 (1.90)	p < 0.001	0.99

### Procedure

A cross-sectional online survey design was used. ASD and TD participants responded to several self-reported measures online, including the ISQ-J. All participants responded to the ISQ-J, MAIA, BPQ-BA, TAS-20, and AQ online. Participants provided text-based informed consent for their participation and publication before survey completion. Participants were instructed that the survey would take approximately 30–40 min of their time and that participation was entirely voluntary, with respondents being able to withdraw at any time.

This study was conducted according to the Declaration of Helsinki. Ethical approval for this study was received from National Rehabilitation Center for Persons with Disabilities (2021-109). The procedure of this study was preregistered on the Open Science Framework (https://osf.io/8epgy).

### Statistical analysis

Statistical analysis was conducted using R (version 3.6.1., by RStudio version 1.2.1335 for Windows; RStudio Team, PBC, Boston, MA). We performed confirmatory factor analyses (CFA) to confirm the validity of the one-factor structure of the ISQ^16^ using the R lavaan package^25^. To judge the model fit, we used the following fit indexes: the standardized root-mean-square residual (SRMR), the root-mean-square error of approximation (RMSEA), and the comparative fit index (CFI) (e.g., Bentler, 1990^26^). To judge the goodness of model fit (one-factor model), we used a cut-off value of less than 0.08 for SRMR, a cut-off value of less than 0.06 for RMSEA, and a cut off value of more than 0.95 for CFI^27^.

As was done in the original study of the ISQ^16^, we also analyzed the relationships among the ISQ-J, MAIA, BPQ-BA, and TAS-20 by calculating correlation coefficients to confirm the convergent validity. In addition, independent samples t-tests were undertaken to compare the group differences in ISQ-J scores between the ASD and TD groups. The complementary analysis was conducted using the AQ score because of the unreliability of formal diagnosis (reliance on participant self-reporting of diagnosis). The participants were split at the 33rd and 66th percentile of AQ scores to create low, middle, and high scoring AQ groups. The low and high scoring groups were used to examine the extent to which ISQ-J could discriminate between groups scoring high and low on autistic traits, as shown in the previous study^16^. Moreover, we performed independent samples t-tests in groups between the AQ threshold cut-off or the above (> 33 points) group or the below (< 1 SD below the mean score) group.

### Ethical approval and consent to participate

The Ethics committees of the National Rehabilitation Center for Persons with Disabilities approved the study, and participants provided full informed consent.

## Results

### Factor analysis of the ISQ

First, we performed CFA to confirm the validity of the one-factor structure of the ISQ-J. The results showed that the goodness of fit for the one-factor model of the ISQ-J (χ^2^(170) = 803.592, p < 0.001, CFI = 0.867, RMSEA = 0.114, SRMR = 0.057). The chi-square test was significant and the comparative fit index (CFI) was close to 0.90, which indicates a moderate level of goodness of fit; and the RMSEA was lower than that in the previous study^19^, although this was more significant than the cutoff score of 0.06, and the SRMR was lower than the cut of score of 0.08.

Then, we calculated the standardized factor loadings to confirm the magnitude of the factor loadings. Table 3 shows the standardized factor loadings for each item. The results revealed that factor loadings of all items were greater than 0.40. The internal consistency measure (Cronbach’s alpha) for the ISQ (α = 0.963) indicated an acceptable level of reliability. Considering these results, we adopted the total score of all 20 items as the total ISQ-J score, following the assumption of the one-factor model in the original study of ISQ^16^.

**Table 3:** Principal axis factor loadings for 20 ISQ items.

Number	Item Stem	Factor loading
1	I have difficulty making sense of my body’s signals unless they are very strong	0.619
2	I tend to rely on visual reminders (e.g. times on the clock) to help me know when to eat and drink	0.467
3	I have difficulty feeling my bodily need for food	0.751
4	I’m not sure how my body feels when it’s a hot day	0.649
5	I find it difficult to describe feelings like hunger, thirst, hot or cold	0.810
6	Sometimes I don’t know how to interpret sensations I feel within my body	0.798
7	If I injure myself badly, even though I can feel it, I don’t feel the need to do much about it	0.690
8	I only notice I need to eat when I’m in pain or feeling nauseous or weak	0.686
9	There are times when I am only aware of changes in my body because of the reactions of other people	0.758
10	I find it difficult to read the signs and signals within my own body (e.g. when I have hurt myself or I need to rest)	0.851
11	I have difficulty understanding when I am hungry or thirsty	0.819
12	I find it difficult to identify some of the signals that my body is telling me (e.g. If I’m about to faint or I’ve over exerted myself)	0.840
13	It is difficult for me to describe what it feels like to be hungry, thirsty, hot, cold, or in pain	0.842
14	I am confused about my bodily sensations	0.798
15	I have difficulty locating injury in my body	0.747
16	Sometimes, when my body signals a problem, I have difficulty working out what the problem might be	0.745
17	I don’t tend to notice feelings in my body until they’re very intense	0.827
18	I find it difficult to put my internal bodily sensations into words	0.814
19	Even when I know that I am hungry, thirsty, in pain, hot or cold, I don’t feel the need to do anything about it	0.753
20	Even when I know that I am physically uncomfortable, I do not act to change my situation	0.779

### Correlations between the ISQ, MAIA, BPQ-BA, and TAS-20

To examine the relationships between the ISQ-J, MAIA, BPQ-BA, and TAS-20, we calculated Pearson’s correlation coefficients between the ISQ-J score and the subscales of MAIA, BPQ-BA, and TAS-20 (Table 4). The correlation analysis showed that the ISQ-J score was positively and significantly correlated with the scores of TAS-20 (r(287) = 0.58, *p* < 0.001) and BPQ-BA(r(287) = 0.29, *p* < 0.001). The strong positive correlation of ISQ-J scores with TAS-20 scores is consistent with the results of a previous study^16^. A significant positive correlation was also found with the BPQ-BA, a measure of awareness of interoceptive sensitivity specifically related to negative emotions, although not as strong as the TAS-20. On the other hand, a significant negative correlation was found between the ISQ-J and the MAIA subscale (attention regulation: r(287) = −0.30, *p* < 0.001, emotional awareness: r(287) = −0.20, *p* < 0.001, self-regulation: r(287) = −0.30, *p* < 0.001, body listening: r(287) = −0.21, *p* < 0.001). This result is consistent with the results of a previous study^16^.

**Table 4:** Correlations between ISQ and convergent validity measures.

		TAS-20	BPQ	MAIA Attention regulation	MAIA Emotional awareness	MAIA Self-regulation	MAIA Body listening
ISQ	ALL (N = 286)	0.58**	0.29**	−0.30**	−0.20**	−0.30**	−0.21**

*p < 0.05, **p < 0.01.

### Group differences in ISQ scores between ASD and TD groups

To test whether the ISQ-J scores were significantly higher in people with ASD than in controls, we conducted independent samples *t*-tests. The results showed significant and extensive differences in ISQ-J scores between ASD and control groups [*t*(89.68) = 7.62, *p* < 0.001, *d* = 1.18]. We also found significant differences in ISQ-J scores between high vs. low autistic traits groups[*t*(186.24) = 6.62, *p* < 0.001, *d* = 0.93] and between above cut-off AQ scores vs. below ones[*t*(95.29) = 10.91, *p* < 0.001, *d* = 2.07]. The results of these independent samples *t*-tests are summarized in Table 5.

**Table 5:** Differences in ISQ scores between ASD and control groups, high vs low autistic traits groups, and above cut-off AQ scores vs below ones.

ASD and control groups				
	ASD (N = 63)	Control (N = 226)	t-test (*p*-value)	Cohen’s *d*
ISQ score (M/SD)	76.78 (25.67)	49.65 (22.19)	< .001	1.18

High autistic traits (> 66th) vs. low autistic traits (< 33rd)				
	High AQ (N = 102)	Low AQ (N = 100)	t-test (*p*-value)	Cohen’s *d*
ISQ score (M/SD)	68.42 (26.16)	47.00 (19.39)	< .001	0.93

Above cut-off AQ scores (> 33 points) vs below ones (< 1SD from average)				
	Above ones (N = 57)	Below ones (N = 44)	t-test (*p*-value)	Cohen’s *d*
ISQ score (M/SD)	80.05 (22.81)	39.57 (14.27)	p < .001	2.07

## Discussion

This study developed and validated a Japanese version of ISQ, a self-rating test for assessing difficulties in processing interoceptive information in individuals with ASD. The analysis investigated the reliability of convergent and discriminant validity in Japanese adolescents and adults with ASD. Our results showed that the internal consistency measure (Cronbach’s alpha) of the ISQ-J indicated an acceptable level of reliability. We also confirmed the convergent validity of the ISQ-J by examining relationships with the TAS-20 and MAIA, as was done in the original study of the ISQ^16^. In addition, comparisons between ASD and control groups showed that the ISQ-J is a reliable instrument for measuring interoceptive confusion in an adult with ASD, as shown in the original ISQ study^16^.

- Validity of the one-factor model
  Our results indicate the validity of the one-factor model of the ISQ-J, consistent with the assumption in the original study^16^. Fiene et al.^16^ designed the ISQ to have one latent factor representing confusion about interoceptive bodily states. They selected 20 items from a 60-item pool based on the results of factor loadings by CFA; item loadings > 0.63 were considered “very good.” Our CFA results showed that all items of the ISQ-J converged on one factor, whose factor loadings were higher than 0.40. Compared to the selection criteria at the item selection, the factor loadings for items 1 and 2 were slightly lower. However, values of factor loadings on items 1 and 2 (0.62 and 0.47) were acceptable and higher than the criteria of other scales that assess the diagnosis of ASD; for instance, the criteria of factor loadings were > 0.30 for the AQ^28^. In addition, the internal consistency of the 20-item ISQ-J in our study (α = 0.963) was an acceptable level, as it was in the original study (α = 0.96)^16^. Therefore, we considered that all the items of the ISQ-J were appropriate for assessing difficulties in processing interoceptive information, which converged on one latent factor.
- Discriminant validity of ISQ-J
  In addition, our results demonstrated the discriminant validity of the ISQ-J with diagnosis-based and traits-based comparisons, as shown in the original ISQ study^16^. Our results showed that scores of the ISQ-J were significantly higher in the ASD group than in the control group. In addition, the discriminant validity of the ISQ-J was also supported by testing relationships with AQ scores. Our results also showed that the scores of the high-AQ and above-cut-off AQ groups were significantly higher than those in the low-AQ groups and below 1 SD from the average group. These results indicate that the Japanese version of the ISQ is a reliable instrument for measuring interoceptive confusion in adults with autism, as demonstrated in the original ISQ study^16^. Moreover, this study revealed that the difference in ISQ-J scores between the group above the AQ cut-off and the group 1 SD below the mean was very large. The results indicate that the perceived strength of awareness of ASD traits might be more related to difficulties with interoceptive processing on the ISQ-J than to the presence or absence of the diagnosis of ASD.
- Convergent validity of ISQ-J

By examining the relationships with the TAS-20 and MAIA, we also showed the convergent validity of the ISQ-J. According to the previous study^16^, we predicted that the ISQ-J would be positively correlated with the TAS-20 Alexithymia scale and negatively correlated with the MAIA, which is composed of items that focus on awareness of the interoceptive information scale in convergent validity analyses of the ISQ-J. Our results replicated the original study’s results^16^; the ISQ-J scores were closely related to the scores of TAS-20 and MAIA. These findings indicate that the confusion of interoceptive information and a trait associated with difficulties noticing one’s own emotions or bodily awareness are strongly linked constructs.

Contrary to our expectations, the scores of the ISQ-J were positively correlated with the scores of the BPQ. One reason for this may be the difference in the question items included in the interoceptive information characteristics. The MAIA is composed of items that focus on awareness of bodily sensations related to neutral-to-positive emotions and asks for an integrated bodily state (e.g., “I notice how my body changes when I feel happy/joyful.”; “I listen to my body to inform myself about what to do.”). In contrast, the BPQ has more questions that focus on awareness of bodily sensations associated with negative emotions and asks for localized bodily states (e.g., “muscle tension in my arms and legs,” “stomach and gut pains”). Therefore, the result of the ISQ would be related to the ease of detecting local body information and the difficulty of noticing integrated body information. This feature may be associated with the weak central coherence (WCC) theory^29^, biased toward local information, making it difficult to integrate and process information in individuals with ASD. Although the WCC theory was constructed based on the characteristics of exteroceptive sensory processing, such as vision, it has recently been proposed that it can be adapted to interoceptive sensory processing in individuals with ASD^13^. The results of the present study support this theory with a self-reported questionnaire.

### Limitations

First, as noted in the previous survey^16^, findings from the ISQ depend on self-report data (including a diagnosis of ASD) to measure validity constructs, which also true for the ISQ-J. However, it is crucial to understand the function of interoceptive processing in individuals with ASD through subjective scales and objective measurement. The previous study pointed out a three-dimensional model of interoception; sensitivity (subjective belief revealed by a self-report), accuracy (objective performance shown by performance on behavioral tests), and awareness (metacognitive accuracy revealed by an insight into performance aptitude)^30^. A behavioral experiment with a self-reported questionnaire revealed reduced interoceptive accuracy (using a heartbeat detection task) and exaggerated interoceptive sensitivity (using a self-reported BPQ) in adults with ASD and that the divergence between the two axes is associated with anxiety in individuals with ASD^14^. In future studies, it would be essential to examine the relationship between the difficulty of interoceptive information processing and behavioral indicators by using the ISQ as well as the BPQ as subjective interoceptive sensitivity.

Second, the sample size of the ASD group in this study is relatively small, and a large percentage of the participants were women. The main reason is that this study used the snow-ball sampling method so that the sample design would be similar to the original ISQ^16^, which made it difficult to survey a large number of people. In addition, this study may not reflect the incidence of ASD because there were more female participants with ASD than male participants. However, the original research^16^ also had a higher percentage of female participants than male participants, so the goal of developing the Japanese version of the ISQ has been achieved. Future research on gender differences in interoceptive confusion may be needed.

Third, since the ISQ is a self-report questionnaire developed for adolescents and adults, it is unclear whether the ISQ can be adapted for use with children or whether it will produce the same results as those for adults. In a behavioral study of interoceptive accuracy, adults with ASD were not significantly different from controls, but children with ASD were significantly lower than controls^31^. Furthermore, in a self-report questionnaire study using the BPQ, adults with ASD showed higher sensitivity than controls^14^, whereas children with ASD showed lower sensitivity than controls^32^. Thus, given that previous studies on interoceptive sensory processing in individuals with ASD have yielded different results for adults and children, the ISQ, when applied to children with ASD, may need to modify the content of its items to match the characteristics of the interoceptive information processing of children of ASD.

Fourth, a previous study that developed a short version of the ISQ (ISQ-8) noted that the original ISQ has duplicate items in the questionnaire and that the reliability coefficient is somewhat low^19^. However, in our Japanese samples, some items with high-reliability coefficients extracted by the ISQ-8 were not as high (e.g., “There are times when I am only aware of changes in my body because of the reactions of other people”), possibly due to cultural differences. Further study is needed to develop an abbreviated version of the ISQ for the Japanese population.

## Conclusions

In summary, the current study demonstrated evidence of the validity of the factor structure and the convergent validity of ISQ-J in a sample comprising Japanese adults. Our results indicate that the ISQ-J can measure difficulties in processing interoceptive information in individuals with ASD. We expect that our development of the ISQ-J will provide a valuable means for assessing confusion of interoceptive information in Japanese individuals with ASD. Moreover, not only in research but also in a clinical setting, assessment with the ISQ-J would be helpful in psychological therapy, occupational therapy, and medical interventions to improve or maintain mental and physical health in individuals with ASD. Further research is needed to explore individual differences in the processing of interoceptive information as well as exteroceptive information and appropriate interventions tailored to the individual sensory processing.

## Data Availability

Data sharing not applicable to this article as no datasets were generated or analyzed during the current study. Please contact the corresponding author (CI) for data requests.

## Abbreviations

ISQ: Interoception Sensory Questionnaire
ISQ-J: Japanese version of Interoception Sensory Questionnaire
BPQ: Body Perception Questionnaire
MAIA: Multidimensional assessment of interoceptive awareness
TAS-20: Toronto Alexithymia Scale
CFA: Confirmatory factor analysis
WCC: Weak central coherence
